# Flagg’s Amalgams

**Published:** 1900-04-15

**Authors:** J. Foster Flagg


### FLAGG'S AMALGAMS.

The *peculiar attribute* of all "sub-marine" amalgams is their property of *sulfiding* when exigencies demand it, and apparently of doing this *in proportion* as this demand is made upon them; so that while in some dentures a given demand causes a brown discoloration of greater or less intensity, in others a greater demand causes an almost immediate discoloration to the blackness of jet.

These results are due to combinations of silver and copper; in most cases more or less controlled by tin, but in cases of absolute necessity of either copper alone or of silver and copper only. This leaves *copper* as the *distinctive* metal for sub-marine amalgam alloys, as *all* dental amalgam alloys contain silver. The formula for "sub-marine" alloy is silver, 60; tin, 33; copper, 7.

The next class of amalgam is "contour," of from 60 to 70 silver, from 25 to 35 tin, from 1 to 3 gold and from 1 to 3 zinc.

This kind of amalgam has done most acceptable service. Its grain is good; it mixes easily with its mercury; it gives ample time for comfortable manipulation, and by "wafering" it is made to set with satisfactory celerity. Its maintenance of color is good when left with that "mat" finish which nearly approximates the color of a tooth, and if it discolors it is usually permanently restored by pumicing and burnishing. But its greatest merit is its reliability.

The third class of amalgam is that known as "facing,"

or "white," a most desirable amalgam *for its purposes*, and which has zinc for its *distinguishing* metal. Its formula is: Tin, 55 parts; silver, 40; zinc, 5.—*J. Foster Flagg, in February Cosmos.*

